# A Sustainable Approach to a Cleaner Production of Antimicrobial and Biocompatible Protein Fibers

**DOI:** 10.3390/polym14153194

**Published:** 2022-08-05

**Authors:** Angela Danila, Mariana Costea, Lenuta Profire, Cristina Mihaela Rimbu, Mihaela Baican, Florentina Lupascu, Simona-Maria Tatarusanu, Bianca-Stefania Profire, Emil-Ioan Muresan

**Affiliations:** 1 Department of Chemical Engineering in Textiles and Leather, Faculty of Industrial Design and Business Management, Gheorghe Asachi Technical University of Iasi, 700050 Iasi, Romania; 2 Department of Kitting and Clothing, Faculty of Industrial Design and Business Management, Gheorghe Asachi Technical University of Iasi, 700050 Iasi, Romania; 3 Department of Pharmaceutical Chemistry, Faculty of Pharmacy, University of Medicine and Pharmacy “Grigore T. Popa”, 700115 Iasi, Romania; 4 Department of Public Health, Faculty of Veterinary Medicine, “Ion Ionescu de la Brad” University of Life Sciences, 700490 Iasi, Romania; 5 Department of Physics, Faculty of Pharmacy, University of Medicine and Pharmacy “Grigore T. Popa”, 700115 Iasi, Romania; 6 R&D Department, Antibiotice, 700548 Iasi, Romania; 7 Department of Internal Medicine, Faculty of Medicine, University of Medicine and Pharmacy “Grigore T. Popa”, 700115 Iasi, Romania; 8 Organic, Biochemical and Food Engineering Department, Faculty of Chemical Engineering and Environmental Protection, Gheorghe Asachi Technical University of Iasi, 700050 Iasi, Romania

**Keywords:** celandine extract, sustainable dyeing, biocompatibility, antibacterial properties, shoe materials

## Abstract

This study presents the production, characterization, and application of celandine (*Chelidonium majus* L.) extracts (aqueous, acidic, alcoholic, and ultrasound) on wool fibers and their characterization. The study aims to obtain an ecologically dyed wool support that possesses biocompatible and antimicrobial activities. The plant extracts were characterized based on pH, total polyphenol content, and berberine content. Ecologically dyed wool supports were characterized based on scanning electron microscopy, levelness index, color measurements, contact angle indirect biocompatibility, and antibacterial analysis. According to the obtained results, celandine extract can be considered a potential candidate for the sustainable dyeing and functionalization of wool fibers.

## 1. Introduction

Environmental problems and the large quantities of non-biodegradable textile waste have urged the textile industry to move towards a sustainable circular economy [1]. The use of bio-based materials is one of the main concerns of today. The use of different plant raw materials and byproducts could be an economically and ecologically useful route leading to sustainable plant-based dyes [2].

Celandine (*Chelidonium majus* L.) is a short-lived hemicryptophyte with a stem length of up to 1 m and is an important medicinal herb used in traditional and folk medicine [3]. *Chelidonium majus* is used for internal and external administration as botanical preparation (extracts, cataplasms) for more than 2000 years, and their benefits for health were first recorded in Antiquity by Dioscorides [4]. Important anti-viral, anti-microbial, anti-inflammatory, and analgesic effects have been observed and sustained by traditional medicine and, to correlate the chemical composition of Chelidonium species with medical applications, the therapeutic effects have been extensively studied in recent decades [5,6].

Different parts of the plant are used in various topical applications, including wart removal, treatment of corns, eczema, and cuts, the treatment of skin wounds, skin irritation, allergic rashes, and dermatitis, in the treatment of boils, and cure blisters, rashes, scabies, skin eruptions, psoriasis, eczema, fungal infections and tumors of the skin [4,7].

The phytochemical composition comprises a wide variety of bioactive constituents from different parts of the plant (leaves, roots, flowers, and latex) that work either apart or synergistically to exert therapeutic effects. Among these, the most studied are alkaloids (berberine, chelidonine, sanguinarine), polyphenols (kaempferol, quercetin), and proteins (chelidocystatin) [6].

The therapeutic action of the celandine plant is largely related to its alkaloid content, which varies between 0.27 and 2.25% (in the aerial parts of the plant) [8]. Although most alkaloids are typically colorless, several of them are colored, like sanguinarine (orange) and berberine (yellow) [9].

Berberine is found in several plants, including *Berberis vulgaris* L., *Thalictrum foliolosum* DC., *Chelidonium majus* L., *Hydrastis canadensis* L., *Phellodendron amurense* Rupr., etc. [10].

**Figure 1:**
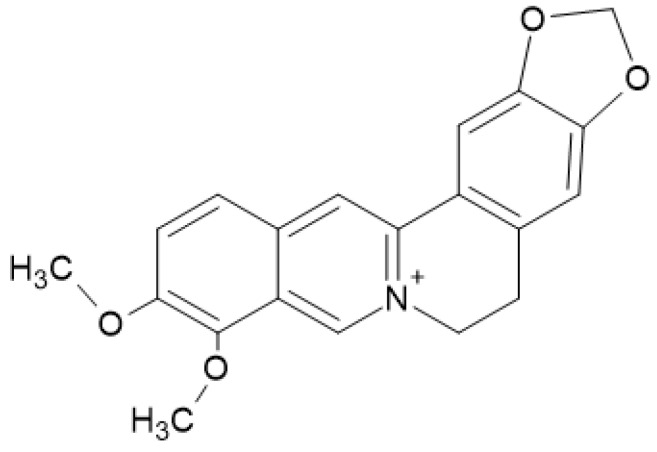
Berberine.

The pharmacologically relevant substances of *Chelidonium majus* L. are isoquinoline alkaloids (components of latex produced in all plant parts). In the celandine plant, five groups of alkaloids (protoberberine, derivatives of phenanthridine, protopine, quinolizidine, and aporphine) can be found. Berberine is among the protoberberine derivatives that accumulate in higher amounts [4]. Berberine (ammonium salt from the protoberberine group of benzylisoquinoline alkaloids) and its derivatives have exhibited potential biological activities that include anti-inflammatory effects [11]. In addition to biological activities, in celandine extract, berberine (Figure 1) has the role of a coloring compound that acts as a basic dye [12].

Although there is little information in the specialized literature about the dyeing of protein fibers with celandine extracts, there are studies that describe textile dyeing with berberine extracted from other plants [13,14,15].

In the present study, the natural dyes extracted from celandine (*Chelidonium majus* L.) were used to obtain biocompatible and antibacterial eco-friendly dyed wool fibers. Three methods of natural plant extraction (aqueous, alcoholic, and ultrasound extraction) were chosen. In this study, citric acid (CA) pretreatment and UV exposure of wool fibers were used to improve the dyeability of wool fabrics with celandine extracts.

## 2. Materials and Methods

### 2.1. Material

**Figure 2:**
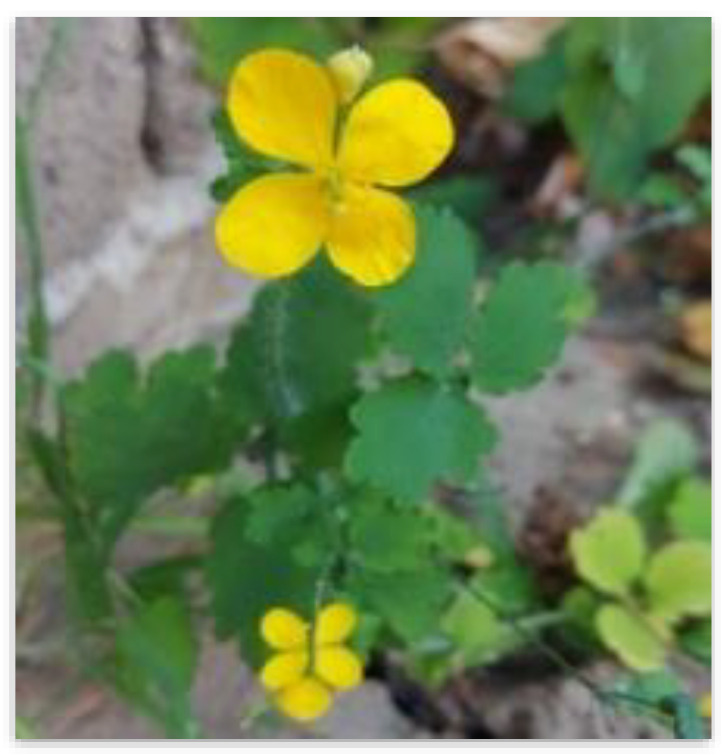
Celandine (*C. majus* L.).

Celandine (*C. majus* L.) leaves and stems were freshly collected from northeastern Romania in October 2021 (Figure 2). The samples were washed with water and dried.

The wool fiber was scoured at 40 °C for 30 min with 2% (*v/v*) commercial Lavotan DSU detergent, rinsed, and dried. For dyeing, scoured wool fibers, wool fibers treated with citric acid, and wool fibers irradiated with UV light were used. Wool fibers were pretreated with 0.1 M citric acid solution at room temperature for 24 h. Pretreated samples were rinsed with distilled water and dried. The wool fibers were subjected to UV exposure for 4 h, using a bactericidal lamp with UV-C ultraviolet light (8 W, 253.7 nm).

All chemicals used in this study were of laboratory grade.

### 2.2. Extraction of Natural Dyes

For this study, 3 methods were chosen for natural plant extraction. For extraction, extracts from dried plants were used.

#### 2.2.1. Aqueous Extraction

##### Aqueous Method

First, 2 g of the vegetal material (leaves and stem of celandine) was boiled with 300 mL of distilled water (duration 60 min). The extract was then filtered. The pH of the extract was recorded.

##### Acidic Method

First, 2 g of the vegetal material (leaves and stem of celandine) was boiled with 300 mL of distilled water with the addition of 1% (*v*/*v*) of glacial acetic acid (duration 60 min). The extract was then filtered. The pH of the extract was recorded.

#### 2.2.2. Alcoholic Method

A 1:1 alcohol/distilled water solution was used for alcoholic extraction. Two grams of the vegetal material were added to the alcoholic solution and maintained for 60 min at 50 °C. The extract was then filtered. The pH of the extract was recorded.

#### 2.2.3. Ultrasound Extraction

First, 2 g of the vegetal material in 300 mL distilled water was extracted in the ultrasound sonicator (Hielscher UP50H) at a frequency of 50 kHz for 20 min (one cycle, amplitude 50%). The extract was then filtered. The pH of the extract was recorded.

### 2.3. pH Measurement of Celandine Extracts

Portable pH measurement instruments (WTW Inolab pHmeter 720, Ihlow, Germany) were used to test the pH of the prepared celandine extracts. The vegetable extracts were stirred shortly before the measurements were performed, and after that, the pH electrode was immersed in the extracts to be measured. The dye extracts were allowed to rest on the sensor until the measured value was displayed.

### 2.4. Total Polyphenol Content

The total polyphenol content was determined by means of the UV-Vis spectrophotometric method, using a GBC Cintra UV-Vis spectrophotometer (Penang, Malaysia).

The calibration curve of gallic acid was determined as previously described in the scientific literature [16,17]. Briefly, 25 mg of gallic acid was dissolved in 25 mL ethanol by sonication. Aliquots of the obtained solution were diluted with 60–70 mL distilled water and mixed with 5 mL of Folin–Ciocalteau reagent (dilution 1:10). After 6 min, 15 mL of Na_2_CO_3_ 7.5% (*m*/*v*) and distilled water in a volume up to 100 mL were added to each sample. The concentration of standard gallic solutions ranged from 0.0625 mg to 1 mg/100 mL. After 2 h of incubation in dark conditions, the absorbance was measured as 720 nm. The calibration curve of gallic acid is the result of the correlation between concentration and absorbance and is y = 60.874x + 0.0326. Aliquots of 1 mL of each *Chelidonium majus* extract were prepared under similar conditions to those employed for the standard solutions. The absorbance was measured at 720 nm and the total content of polyphenols was expressed on the calibration curve.

### 2.5. Berberine Content

The quantitative analysis of berberine in the celandine extracts was performed by means of the UV-Vis spectrophotometric method, using a GBC Cintra UV-Vis spectrophotometer. The standard solution of berberine was prepared as previously described [18,19]. Briefly, 1 mg/mL stock solution was prepared by dissolving 10 mg of berberine hydrochloride in 10 mL methanol. Furthermore, the stock solution was diluted with methanol to obtain standard solutions of berberine with concentrations ranging from 2 to 10 µg/mL, and the absorbance was measured at 426 nm. The calibration curve of berberine is the result of the linear correlation between concentration and absorbance and is y = 0.1551x − 0.0237. Then, 1 mL aliquots of each *Chelidonium majus* extract were prepared under the same conditions as the standard solutions. The absorbance was measured at 426 nm and the concentration of berberine was expressed on the calibration curve.

### 2.6. Dyeing

**Figure 3:**
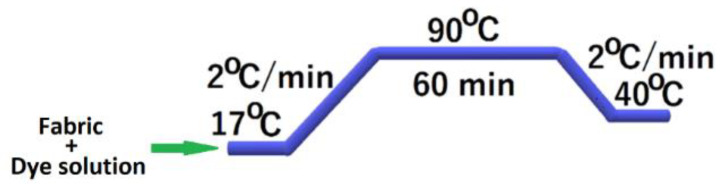
Wool dyeing diagram.

The wool fabric sample was first immersed in distilled water at room temperature and then in the celandine extracts (Figure 3). All of the dyeing experiments were carried out with a material-to-liquor ratio of 1:70 at the initial pH of the celandine extracts at 90 °C for 1 h (heating rate 2 °C/min). A Polycolor beaker dyeing system type P, (R.D. Mathis Company, Signal Hill, CA, USA), was used for dyeing. The samples were shaken at 16 rotations per minute. After dyeing, the dyed wool samples were washed with a non-ionic surfactant at 40 °C for 30 min, rinsed with warm water, and air-dried.

### 2.7. Scanning Electron Microscopy (SEM)

The SEM images of wool fibers (raw and dyed) were obtained using a HITACHI Tabletop Microscope (Nitech, Krefeld, Germany) with a magnification of 2000× at an acceleration voltage of 5.0 k under a SE (secondary electron) detector [20].

### 2.8. Determination of Levelness Index

The levelness of dyed wool samples was calculated by measuring the reflectance values at 10 nm intervals between 400 and 700 nm. Each sample was measured three times by randomly changing the measuring point. The relative unleveled index (RUI) was calculated using Equation (1):

(1) $$\mathrm{RUI}=\overset{700}{\underset{400}{\sum }}\frac{S_{\lambda }}{R_{\lambda }}\cdot V_{\lambda }$$

where *R_λ_*—mean reflectance values for each wavelength; *S_λ_*—standard deviation of reflectance values; *V_λ_*—photopic relative luminous efficiency function [21]. The interpretations of the RUI values are listed in Table 1 [22].

### 2.9. Color Measurements

SF 300, DATA COLOR spectrophotometer (Rotkreuz ZG, Switzerland) was used to quantify the color coordinates CIE (L*, a*, b*) and color strength (*K/S*) after dyeing. The samples were measured five times and the average values were recorded. The *K/S* values were assessed using the Kubelka–Munk Equation (2):

(2) $$\frac{K}{S}=\frac{{\left(1-R\right)}^{2}}{2R}$$

where *K* is the absorption coefficient, *S* is the scattering coefficient, and *R* is the reflectance of the samples at the maximum absorption wavelength [22]. The conditions used in the experiment were the standard illuminant D65 and the 10° observer.

### 2.10. Contact Angle and Indirect Biocompatibility Studies

To study the wettability of the wool fabrics (raw and dyed), at least 10 replicates were performed for each sample, using three pure liquids (distilled water, formamide, and diiodomethane). The fabric samples (1 cm × 5 cm) were kept at room temperature (20 ± 2 °C) for 24 h and were then placed on a tabletop of the Camplus Micro equipment. A droplet of each of the liquids used (distilled water, formamide, and diiodomethane) was placed vertically onto the sample surface using a dedicated Hamilton syringe with a fixed steel needle for each liquid. Each measurement was recorded at different points of the samples. The contact angles and the free surface energy were determined as previously described [23,24,25].

### 2.11. Antimicrobial Analysis

The Kirby-Bauer diffusion method, adapted for the study of wool samples, was used to evaluate antimicrobial activity. The principle of this technique is to bring the test samples into contact with different strains of bacteria, and the bioactive principles active in the samples diffuse into the environment and inhibit microbial culture.

The tests were performed with the reference bacterial strains (American Type Culture Collection) Staphylococcus aureus ATCC 25923 (a Gram-positive strain) and Escherichia coli ATCC 25922 (a Gram-negative strain). For this, microbial suspensions with a 0.5 McFarland Standard equivalent to approximately 1.5 × 10^8^ bacterial cells/mL, were obtained from 24 h cultures. A 1 mL volume of the bacterial suspension was distributed into Petri dishes over which Mueller Hinton Agar Medium (Bio-Rad Laboratories, Dialab Solution, Bucharest, Romania) was added. The wool samples were weighed (40 mg) and distributed with equal spacing on the surface of the culture medium before being incubated at 37 °C for 24 h (Incubator Binder BD23, Tuttlingen, Germany).

Antimicrobial activity was qualitatively assessed by measuring the area of microbial inhibition formed from the edge of the sample to the edge of the zone of inhibition. The samples were tested in triplicate.

## 3. Results and Discussion

### 3.1. Analysis of Plant Extracts Obtained

**Figure 4:**
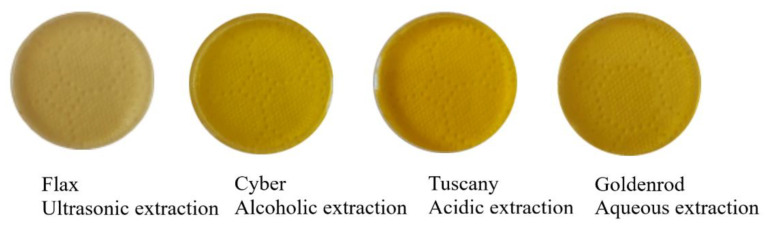
Shades of pure celandine extracts.

Generally, yellow colors are the most common color obtained by dyeing with natural dyes [26]. Following the extraction processes, different shades of yellow were obtained, as shown in Figure 4.

Extraction efficiency depends on the polarity of the solvents (water 1, ethanol 0.654, acetic acid 0.648) and increases with solvent polarity [27]. In addition to the nature of the solvent and pH, the temperature is another factor that influences the extraction efficiency. The extraction rate can be increased by increasing the temperature. An increase in temperature results in an increase in diffusivity and a decrease in the surface tension of the solvent [28].

**Figure 5:**
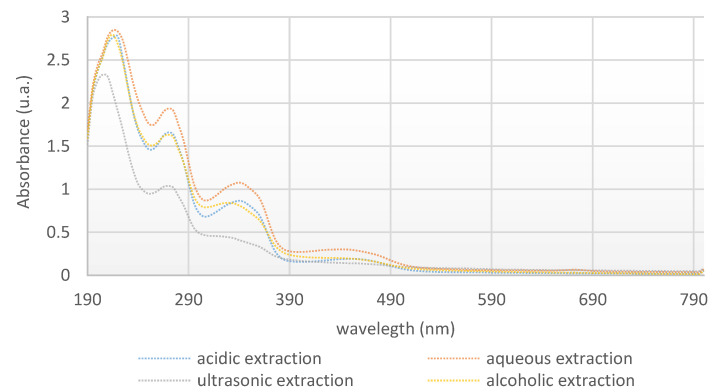
UV-Vis spectra of celandine extracts.

The extracted solutions were characterized using UV–Visible spectrophotometry. The UV-Vis spectra of the solutions at wavelengths from 200 to 800 nm are shown in Figure 5.

UV spectra are used for the confirmation of the presence of berberine in the celandine extracts. The absorption spectra reveal four bands [29] with maxima around 205–220 (literature data [30]: 206–213), 270–275 (literature data [31]: 265–270), 330–335 (literature data [29,32]: 330,350), and 440–445 nm (literature data [29,33]: 400–412, 430).

### 3.2. pH Values

Because wool dyeing was performed with the celandine extracts at their initial pH, their pH plays a significant role (Table 2).

The pH of the analyzed extracts varies from a strongly acidic pH (4.26) to a weakly alkaline pH (7.37). Decreasing the pH reduces the tendency to wick and degrade the wool fiber and increases its dyeing capacity. The action of the acid consists in the fact that by its dissociation, a portion of the hydrogen ions bind to the amino groups due to the amphoteric character of the wool, which, as a consequence, results in the wool having an electropositive charge, thereby increasing its affinity for acid dye ions. Hydrogen ions easily penetrate inside the fiber and bind to the basic grouping of the wool.

### 3.3. Total Polyphenol Content

**Figure 6:**
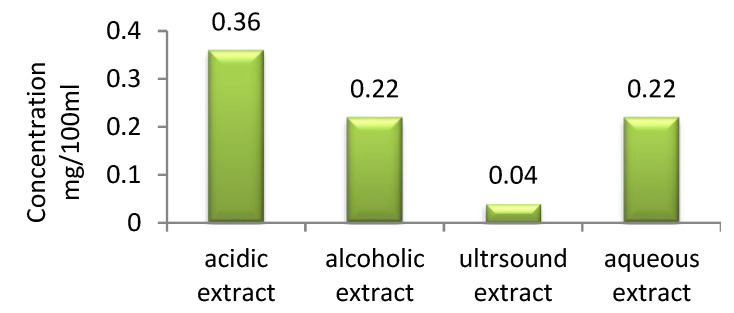
Total polyphenol content.

The total polyphenol content of the celandine extracts, expressed as gallic acid mg/100 mL, is graphically presented in Figure 6.

The obtained celandine extracts presented different total polyphenol contents. The highest content (0.36 mg/100 mL) was observed for the extract in acid medium, and the lowest (0.04 mg/100 mL) for extract obtained by sonication, while the extracts obtained in hydro-alcoholic medium and water had the same polyphenol content (0.22 mg/100 mL). These results support the key role played by the extraction method, which is responsible for the solubilization of the polyphenols in the extraction medium. A possible explanation for the maximum content of total polyphenols being observed in the acid extract is that the addition of acid leads to increased solvent polarity and degradation of the plant’s cellulosic cell matrix, thus improving the solvent accessibility inside the matrix as wel as dye extraction [34].

### 3.4. Berberine Content

**Figure 7:**
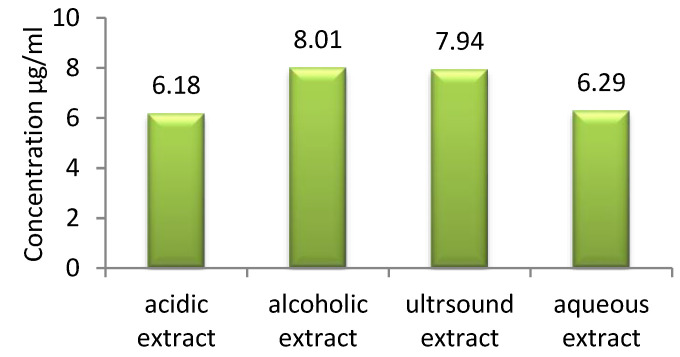
Berberine content.

The berberine content of the celandine extract samples is graphically presented in Figure 7.

It was observed that the differences in the berberine contents of the extracts were smaller than those in total polyphenol content. Sensitivity to heat is one of the major challenges for berberine extraction [35]. Thus, the highest berberine content was observed in the hydro-alcoholic extract and that obtained by sonication, which contained almost 25% more berberine than the extracts prepared in the acidic medium and water.

### 3.5. Dyeing Mechanism

**Figure 8:**
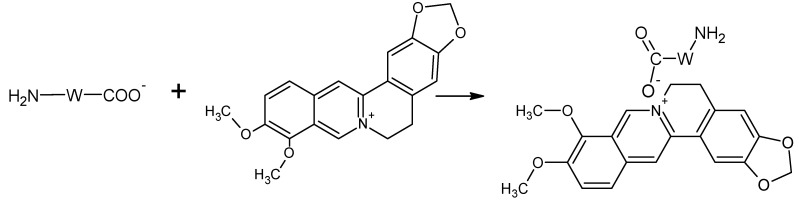
Proposed dyeing mechanism for untreated wool.

**Figure 9:**
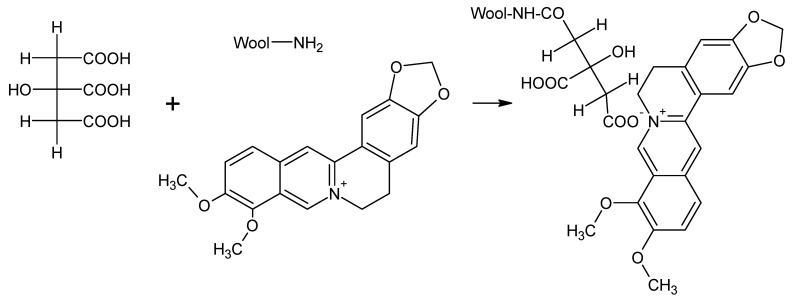
Proposed dyeing mechanism for citric acid pretreated wool.

Among natural dyes, only alkaloid dyes act as basic dyes, in which there is a grouping with a quaternary nitrogen atom (>N^+^-). The nitrogen atom from alkaloids is positively charged and can bind to carboxylic groups from wool fibers [36]. With slightly acidic pH, and especially at neutral pH, berberine can partially bind to various substances as a basic dye (Figure 8). Naturally, at acidic pH, berberine dye solubility is significantly increased [37]. Citric acid facilitates the formation of crosslinks with wool polypeptide chains, and is capable of forming amide linkage with the -NH_2_ groups of wool (Figure 9) [38]. The chemical change caused by the UV treatment is due to surface oxidation of cysteine (disulfide bonds) and induces changes in the dyeing properties of the wool (due to the increased diffusion coefficient of the dyes in the treated wool fibers) [39].

### 3.6. Scanning Electron Microscopy

**Figure 10:**
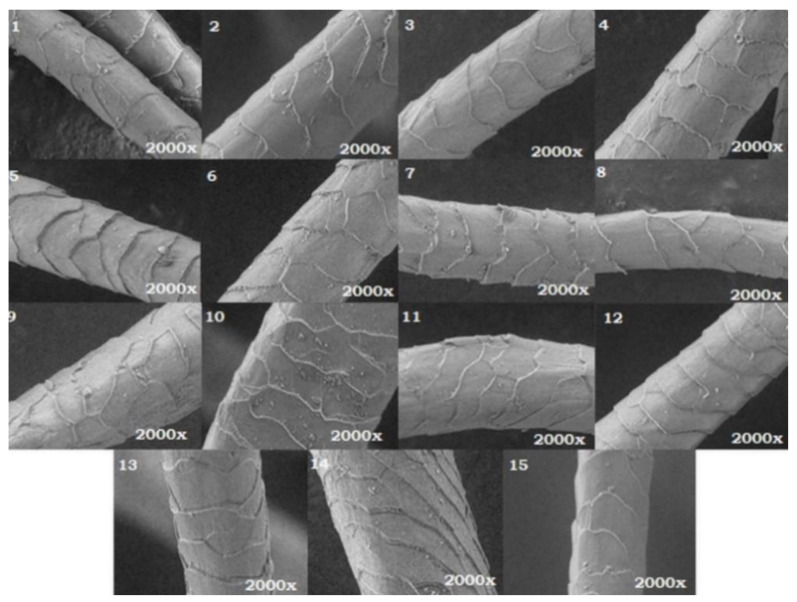
Scanning electron micrograph of (**1**) wool dyed with aqueous extract, (**2**) wool dyed with ultrasonic extract, (**3**) wool dyed with alcoholic extract, (**4**) wool dyed with acidic extract, (**5**) UV-irradiated wool dyed with aqueous extract, (**6**) UV-irradiated wool dyed with ultrasonic extract, (**7**) UV-irradiated wool dyed with alcoholic extract, (**8**) UV-irradiated wool dyed with acid extract, (**9**) wool treated with citric acid and dyed with aqueous extract, (**10**) wool treated with citric acid and dyed with ultrasonic extract, (**11**) wool treated with citric acid and dyed with alcoholic extract, (**12**) wool treated with citric acid and dyed with acid extract, (**13**) wool treated with citric acid, (**14**) UV-irradiated wool, and (**15**) untreated wool.

The results of the SEM analysis of the studied wool fibers are presented in Figure 10.

The possible change in the morphological structure of the wool fibers due to pre-treatments (by UV irradiation and citric acid treatment) or dyeing with celandine extracts was investigated by comparing it with that of untreated fibers at 2000× magnification. It was observed that the surface structure of the wool fibers was not affected by UV radiation. For this reason, the physical or mechanical properties of wool fibers remained unaffected by pretreatments or dyeing [1].

### 3.7. Color Measurements

**Figure 11:**
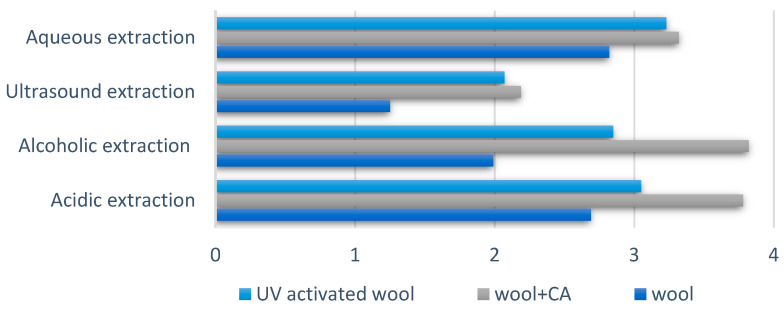
Color strength at 400 nm (maximum absorption).

The color strength of dyeing wool fibers was measured in the range of 400–700 nm under the illuminant D65. A higher *K/S* value represents better color strength of the dyed wool samples. Depending on the pretreatment applied to the wool samples, color strength values decrease in the following order: wool pretreated with citric acid > UV activated wool > untreated wool (Figure 11). Citric acid facilitates the formation of permanent crosslinks with wool polypeptide chains. The Carboxyl group in acid increases the color strength of the dyed wool fibers. Wool irradiated with ultraviolet radiation absorbs dyes from the dye bath faster. This is explained by the fact that the cuticular layer (which is a barrier to the absorption of dyes) of the fiber is attacked during the ultraviolet irradiation process.

The lowest values of color strength are those of the sample dyed by sonication. This indicates that the color yield of the sample batch-dyed by sonication was lower, with lower dye absorption in the fiber.

The CIE (L*—lightness of the color; a*—red/green balance of color; b*—yellow/blue balance of color; C*—chromaticity; *h*—hue angles) color coordinates [21] are listed in Table 3.

The application of celandine extracts on wool fibers produced yellow shades, possessing hue angles ranging between 79.07 and 83.31 for untreated samples, between 79.52 and 85.20 for wool samples pretreated with citric acid, and between 80.11 and 86.96 for UV-activated wool samples.

The decrease in lightness was attributed to more dye being absorbed into the wool fibers, producing a darker shade. Dyeing with ultrasound extract is the lightest.

According to Table 3, all samples have positive values for the *a* and *b* parameters, which confirms that their color is in the range of red–orange–yellow.

### 3.8. Determination of Levelness Index

Table 4 shows the RUI values of dyed wool fibers. The best RUI values were obtained for wool samples dyed with the vegetal dye extracted in acetic acid (Table 4).

The dyeing of the wool with cationic dyes is done in neuter or acidified with acetic acid baths. Acidification slows down the dye by increasing the solubility of the dye, thus preventing the dissociation of the carboxylic groups of the wool. Slowing down the dyeing means better conditions for color levelness.

### 3.9. Contact Angle and Indirect Biocompatibility

The rough surface of the wool fibers and the fabrics manufactured from wool, which was demonstrated using SEM microscopy, is the main challenge in reaching a precise and accurate contact angle measurement [40]. In order to achieve greater accuracy, for this test, we used wool fabric instead of wool fibers. The wool fabrics were pretreated and dyed with the celandine extracts under the same conditions as those used for the wool fibers. The values of contact angle were measured using three different liquids (water, formamide, and diiodomethane), and the corresponding standard deviations for each measurement are presented in Table 5.

The values recorded for the raw wool fabrics, approximately 120°, were similar to other data reported in the literature [40].

**Figure 12:**
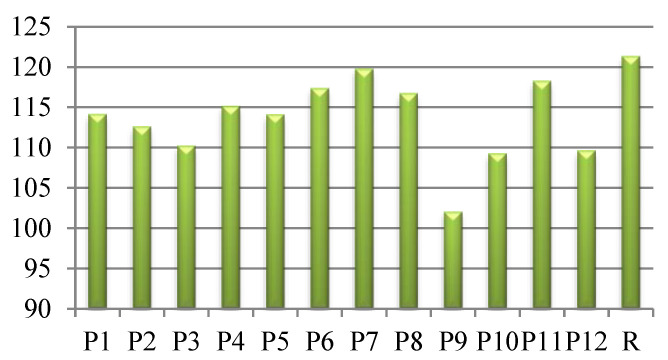
Contact angle values of dyed wool (P1–P12) and raw (R) fabrics.

As can be seen in Figure 12, all dyed fabrics had a smaller hydrophobic character (smaller contact angles with water) when compared with the raw wool fabrics (especially samples P3, P9, P10, P12).

This behavior could be explained by the hydrophilic groups present in the *Chelidonium majus* extracts used as dyeing agents, mainly of the polyphenol hydroxyl groups. In addition, the pre-treatment applied to the raw fiber before dyeing (UV exposure and citric acid treatment) could modify not only the fiber morphology but also the chemical reactivity on the outer layer of the wool fiber.

Based on the contact angle values, the surface free energy (SFE) of the sample was calculated using Young’s complete Equation (3), which is the result of both apolar and polar interactions between solids and liquids [41].

(3) $$\left(1+cos\theta \right)\gamma_{l}=2\left(\sqrt{\gamma_{s}^{LW}\gamma_{l}^{LW}}+\sqrt{\gamma_{s}^{+}\gamma_{l}^{-}}+\sqrt{\gamma_{s}^{-}\gamma_{l}^{+}}\right)$$

where

cosθ = cosine of the measured contact angle;

$\gamma_{l}$ = surface free energy for the liquid;

$\gamma_{s}^{LW}\;,\;\gamma_{l}^{LW}$ = dispersive component of surface free energy for solid (*s*) and liquid (*l*);

$\gamma_{s}^{+}\;,\;\gamma_{l}^{-}$ = polar components of surface free energy (the donor and, respectively, the acceptor of electrons) for solid (*s*) and liquid (*l*).

**Figure 13:**
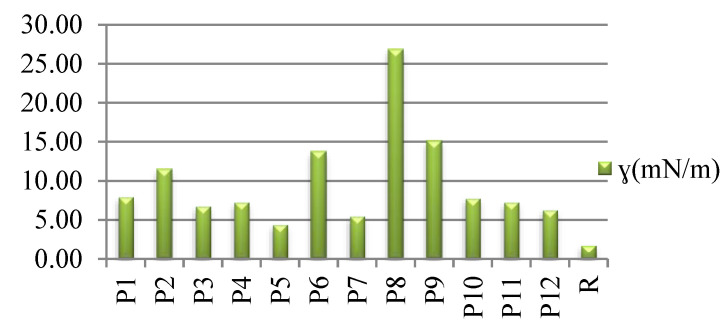
Polar components of surface free energy for wool fabrics, dyed (P1–P12) and raw (R).

As can be seen in Figure 13, all of the samples exhibit higher SFE than the untreated wool fabrics. The highest value was obtained for the P8 sample, which was pre-exposed to UV radiation before dyeing with the *Chelidonium* extract prepared by the acetic acid method.

SFE results from the interactions between the molecules that are present on the outer layer of the wool fibers, and provides information about how the fabrics will behave when coming into contact with the human body [25,42]. The data of an in vivo study evaluating the influence of SFE on fabrics made from cotton and polyester strongly suggest that thermal stress under dry and warm conditions was significantly reduced in subjects who wore clothes and shoes manufactured from fabrics with higher hydrophilic properties compared with the group who wore hydrophobic equipment [42]. With respect to fibers and polymers with medical applications, important attention has been devoted to the biocompatibility of the materials with the human body. One of the primary sources of information in this regard is studies on material surfaces. The physicochemical properties of a surface, such as wettability and roughness, are related to interactions that occur when the material comes into contact with body fluids (water and protein adsorption and cell adhesion) [43,44]. Depending on the intended use (implant, patches, ocular lens), the interfacial tension of a biomaterial coming into contact with biological fluids will have different values [40]. For a biomaterial that is designed to be used as a tissue engineering scaffold, the interfacial tension must be in the range of 1–3 mN/m for biocompatibility to be achieved [45,46].

**Figure 14:**
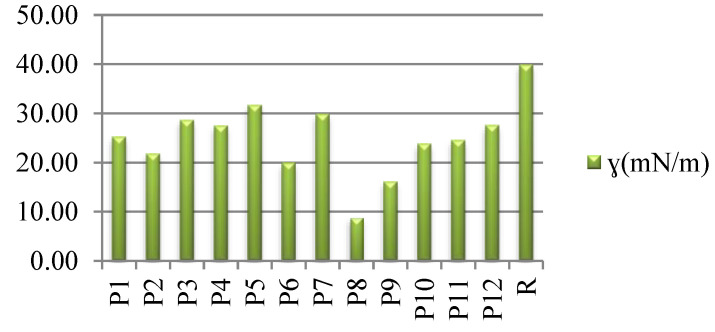
Interfacial tension of wool fabrics, dyed (P1–P12) and raw (R).

Hence, we determined the influence of the dyeing process on the biocompatibility of the dyed fabrics, with raw fabrics acting as a reference, by using the values of SFE as an indirect method. All dyed samples presented decreased values for interfacial tension with the biological fluids, which indicates a tendency towards increased biocompatibility (especially in the case of the P8 sample) when compared with the raw fabrics (Figure 14).

Our results reveal notable differences regarding the surface properties of the analyzed samples as a result of applied pre-treatments and the dyeing process. Better hydrophilicity was expected for the fiber dyed with acidic extract, as it had the highest total polyphenol content, which endows the fibers with a greater number of hydroxyl groups. Nevertheless, it can be seen that it is actually P9 (pre-treated with citric acid and dyed with aqueous extract of *Chelidonium majus*) that possessed the best wettability, while P8 (exposed to UV radiation and then dyed with acidic extract) exhibited the best biocompatibility, as its SFE was the highest. These differences can be explained by the synergistic effect between pre-treatments and the dyeing extracts used.

Therefore, the dyeing of wool fibers with natural products obtained by different methods has a positive impact on fabrics’ behavior in terms of their hydrophilicity, and this is interconnected with the surface properties of the raw fiber.

### 3.10. Antimicrobial Analysis

Insoles are products that frequently come into contact with the skin of the feet. The microbiota of the skin is very complex, and is mainly colonized by Gram-positive and Gram-negative bacteria. Its composition depends on the physiology of the skin, which is mainly related to the degree of hydration and the sebaceous state of the anatomical region. On the other hand, many human infections are caused by bacteria, such as diabetic foot infections [47,48]. Turhan V. et al. identified in their research the bacterial organisms that are found in the case of diabetic foot infections. The most commonly isolated bacteria were *Enterobacter species* (7.1%), *Escherichia coli* (7.1%), *Enterococcus species* (11.5%), *Staphylococcus aureus* (16.7%), and *Pseudomonas species* (29.8%) [49].

**Figure 15:**
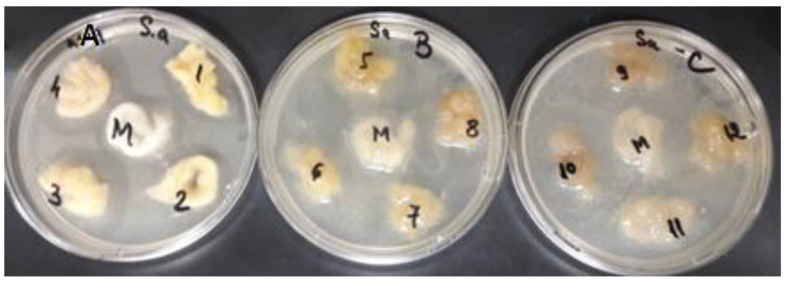
Antimicrobial activity of wool samples against *Staphylococcus aureus* ATCC 25923: A—wool samples treated with citric acid; B—UV-irradiated wool samples; C—untreated wool samples; 1—wool dyed with aqueous extract, 2—wool dyed with ultrasonic extract; 3—wool dyed with alcohol extract; 4—wool dyed with acid extract, M—control.

**Figure 16:**
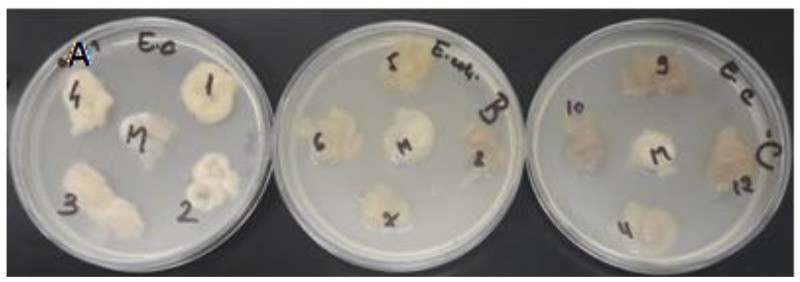
Antimicrobial activity of wool samples against *Escherichia coli* ATCC 25922: A—wool samples treated with citric acid; B—UV-irradiated wool samples; C—untreated wool samples; 1—wool dyed with aqueous extract, 2—wool dyed with ultrasonic extract; 3—wool dyed with alcohol extract; 4—wool dyed with acid extract, M—control.

For antimicrobial activity testing, all wool samples were tested simultaneously on two reference bacterial strains: *Staphylococcus aureus* ATCC 25923 and *Escherichia coli* ATCC 25922. The antimicrobial potential was determined by measuring the microbial zone of inhibition, representing the area in which the microbial culture did not develop (Figure 15 and Figure 16).

**Figure 17:**
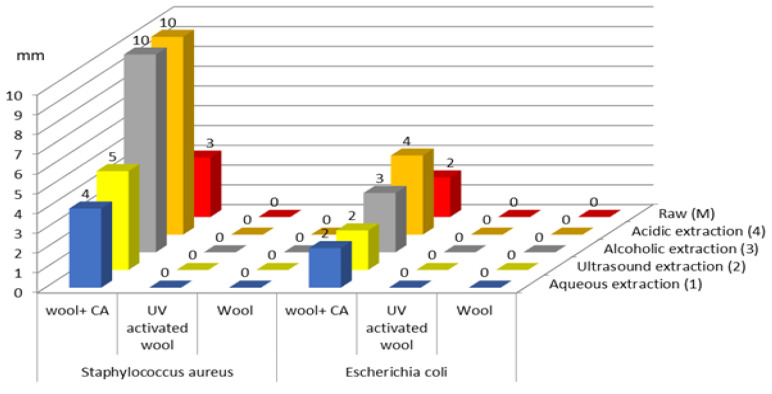
Zones of bacterial inhibition of treated wool fibers.

After testing the antimicrobial activity of the wool samples, it can be seen that the results were influenced by both the type of treatment of the wool fibers and the type of extract used (Figure 17).

Antimicrobial activity was reported only in wool samples treated with citric acid, and was reported against both bacterial strains tested.

For *Staphylococcus aureus*, the most significant antimicrobial effect was obtained from the citric acid-treated fibers dyed with alcoholic extract (10 mm) and acidic extract (10 mm). For *Escherichia coli*, the microbial susceptibility profile for citric acid-treated fibers showed the same variations in inhibition zones, with the highest antimicrobial activity being observed for the wool fibers dyed with acidic extract (4 mm) and alcoholic extract (3 mm).

Comparing the results obtained with the inhibition zones of the controls (citric acid-treated and undyed wool fibers) for both *Staphylococcus aureus* (3 mm) and *Escherichia coli* (2 mm), it can be seen that the antimicrobial effect is potentiated by the bioactive compounds from the plant extracts with which the samples were dyed.

The antimicrobial activity of the control sample can be explained because it was treated with citric acid, and its antimicrobial effect is known [50], although the exact mechanism of action is not known. One explanation would be the ability of citric acid to disrupt the electron transport system and cross the bacterial cell membrane unhindered when in a non-dissociated state (CAH3), whereupon it dissociates in the bacterial cytoplasm into CA anions and protons, leading to acidification of the intracellular medium, thus affecting the enzymatic activity and the molecular structure of bacterial DNA [50,51].

The antimicrobial activity of the wool samples treated with citric acid was enhanced by the bioactive compounds contained in the extracts of *Chelidonium majus* L. Some studies have shown that both alcoholic and aqueous extracts of *Chelidonium majus* L. are rich in alkaloids (chelidonine, chelerythrine, sanguinarine, berberine, coptysine, allocryptine, protopine, etc.), and have potent antimicrobial activity against both Gram-positive (*Staphylococcus aureus*) and Gram-negative (*Escherichia coli*) bacteria [4,52]. Organic insoles made from natural wool fibers remain at the forefront of this type of product as well as being popular in terms of consumer preference. The enrichment of the raw material with antimicrobial properties by natural dyes from *Chelidonium majus* L leads to the maintenance of local foot health by reducing microbial proliferation, which is generally favored by sweat.

## 4. Conclusions

In our study, the wool fabrics were dyed using *Chelidonium majus* extract, which is used as a functional material with antibacterial properties in shoe manufacturing. We studied an environmentally friendly approach for producing biocompatible, antimicrobial, and dyed materials. Dyeing with celandine extracts of wool fabric was extremely beneficial, and did not require the use of any hazardous substances during the treatments. The results demonstrated that pretreatments with citric acid and UV-irradiated wool fibers improved dye uptake, compared with untreated samples. This increase became more pronounced for pretreatment with citric acid. The study showed that samples pretreated with citric acid and dyed with celandine extracts had an antibacterial effect on bacteria *S. aureus* and *E. coli* growth. All dyed samples are biocompatible.

## Figures and Tables

**Table 1:** Interpretation of the RUI values.

RUI	Visual Appearance of Levelness
<0.2	Excellent
0.2–0.49	Good
0.5–1.0	Poor
>1.0	Bad

**Table 2:** pH values of celandine extracts.

	Alcoholic Extract	Acidic Extract	Aqueous Extract	Ultrasound Extract
pH	7.37	4.26	7.03	7.05

**Table 3:** CIE-L*a*b* values, and shades of dyed wool samples.

		L*	a*	b*	C*	*h*	Sample Shade
Untreated wool	Aqueous extract (P1)	66.98	4.79	40.81	41.09	83.31	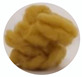
	Ultrasound extraction (P2)	67.62	4.09	22.31	22.68	79.61	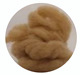
	Alcoholic extraction (P3)	69.10	3.99	25.56	25.87	81.14	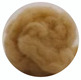
	Acidic extraction (P4)	66.36	5.07	26.24	26.72	79.07	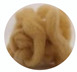
UV activated wool	Aqueous extract (P5)	67.68	4.91	43.13	43.41	83.51	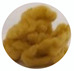
	Ultrasound extraction (P6)	77.02	1.68	31.59	31.64	86.96	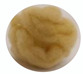
	Alcoholic extraction (P7)	63.35	2.55	30.21	30.32	85.18	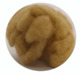
	Acidic extraction (P8)	62.79	3.97	22.79	23.13	80.11	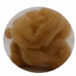
wool + CA	Aqueous extract (P9)	62.20	5.28	28.56	29.04	79.52	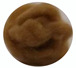
	Ultrasound extraction (P10)	74.06	3.12	37.12	37.25	85.20	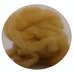
	Alcoholic extraction (P11)	65.23	4.39	35.29	35.56	82.91	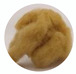
	Acidic extraction (P12)	63.42	5.01	27.21	27.67	79.57	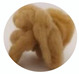

**Table 4:** The relative unleveled indices (RUI) of dyed wool fibers.

	Acidic Extraction		Alcoholic Extraction		Ultrasound Extraction		Aqueous Extraction	
Wool + CA	0.49	Good	0.89	Poor	0.53	Poor	0.60	Poor
UV activated wool	0.31	Good	0.58	Poor	0.37	Poor	0.70	Poor
wool	0.24	Good	0.24	Poor	0.59	Poor	0.28	Poor

**Table 5:** The values of contact angle.

	Ꝋ _water_	stdev_Ꝋ__water_	Ꝋ _formamide_	stdev_Ꝋ__formamide_	Ꝋ _diiodomethane_	stdev_Ꝋ__diiodomethane_
P1	114.14	3.02	116.14	4.21	114.14	8.48
P2	112.59	7.62	118.64	2.57	118.12	6.84
P3	110.21	6.95	112.57	8.47	119.12	4.33
P4	110.64	4.95	117.93	4.75	118.11	3.96
P5	114.10	4.84	112.32	3.78	113.18	5.49
P6	117.35	2.73	125.00	3.39	119.58	4.23
P7	119.75	5.07	106.14	3.42	115.05	5.01
P8	109.92	6.81	125.36	3.78	108.41	4.87
P9	102.02	6.80	107.96	4.20	110.59	4.21
P10	109.27	9.85	109.27	3.27	108.11	3.00
P11	118.26	6.35	116.79	3.13	107.90	3.19
P12	109.62	4.04	109.62	2.97	112.59	6.05
Untreated wool	121.35	5.63	115.24	2.80	112.43	5.78
